# Supplementary material for: Splice-Junction-Based Mapping of Alternative Isoforms in the Human Proteome
Source: Cell Rep. Author manuscript; Available in PMC 2020 Jan 15. (PMC6961840; doi:10.1016/j.celrep.2019.11.026)
Supplement: 2 [file NIHMS1546469-supplement-2.zip › DF1/PXD010154/Prostate/Prostate_16_SZT2_AEGTNGWHAAPSGAPRPHLPVSAFSVDLSPR.pdf]

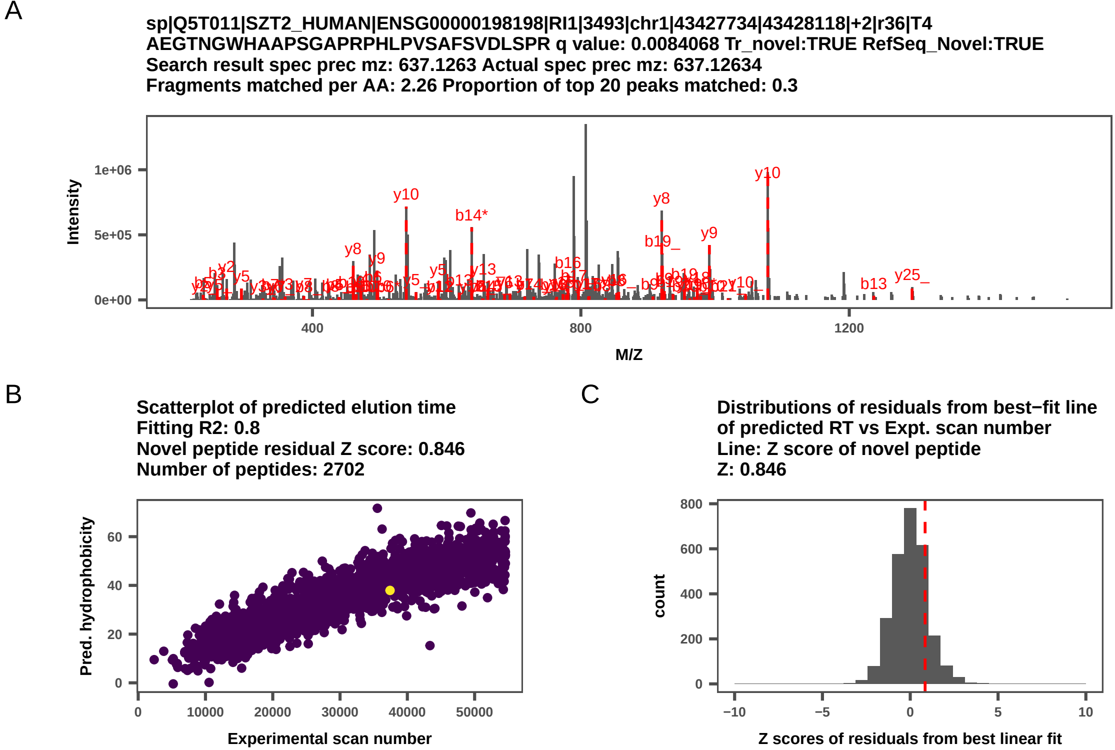
A
sp|Q5T011|SZT2_HUMAN|ENSG00000198198|RI1|3493|chr1|43427734|43428118|+2|r36|T4
AEGTNGWHAAPSGAPRPHLPVSAFSVDLSPR q value: 0.0084068 Tr_novel:TRUE RefSeq_Novel:TRUE
Search result spec prec mz: 637.1263 Actual spec prec mz: 637.12634
Fragments matched per AA: 2.26 Proportion of top 20 peaks matched: 0.3
Intensity
1e+06
5e+05
0e+00
y10
y8
b14*
y10
y8
y9
b19_
y9
y13
b16
y5
b13
y25_
400
800
1200
M/Z
B
Scatterplot of predicted elution time
Fitting R2: 0.8
Novel peptide residual Z score: 0.846
Number of peptides: 2702
Pred. hydrophobicity
60
40
20
0
0
10000
20000
30000
40000
50000
Experimental scan number
C
Distributions of residuals from best−fit line
of predicted RT vs Expt. scan number
Line: Z score of novel peptide
Z: 0.846
count
800
600
400
200
0
−10
−5
0
5
10
Z scores of residuals from best linear fit
